# Investigating the effect of augmented reality-based virtual patient training on occupational therapy students’ clinical decision-making: A quasi-experimental study

**DOI:** 10.1371/journal.pone.0340759

**Published:** 2026-02-04

**Authors:** Farshid Chahartangi, Nahid Zarifsanaiey, Manoosh Mehrabi, Bahareh Zeynalzadeh Ghoochani, Nahid Sharifzadeh

**Affiliations:** 1 Department of E-learning, Virtual School, Comprehensive Centre of Excellence for e-Learning in Medical Sciences, Student Research Committee, Shiraz University of Medical Sciences, Shiraz, Iran; 2 Department of E-learning, Virtual School, Comprehensive Centre of Excellence for e-Learning in Medical Sciences, Shiraz University of Medical Sciences, Shiraz, Iran; 3 Department of E-learning, Virtual School, Comprehensive Centre of Excellence for e-Learning in Medical Sciences, Shiraz University of Medical Sciences, Shiraz, Iran; 4 Department of Occupational Therapy, School of Rehabilitation Sciences, Shiraz University of Medical Sciences, Shiraz, Iran; 5 Department of E-learning, Virtual School, Comprehensive Centre of Excellence for e-Learning in Medical Sciences, Shiraz University of Medical Sciences, Shiraz, Iran; Hamadan University of Medical Sciences School of Dentistry, IRAN, ISLAMIC REPUBLIC OF

## Abstract

Technology-enhanced education is an essential element of modern universities, improving clinical decision-making, and student performance. This study aimed to design and evaluate an augmented reality-based virtual patient (VPAR) educational model to enhance clinical decision-making in upper limb nerves among occupational therapy students. This quasi-experimental study conducted from January to April 2025 with 44 occupational therapy students from Shiraz and Arak Universities of Medical Sciences. Classes were assigned by pre-existing cluster allocation to the intervention group (VPAR, n = 24) or the control group (traditional education, n = 20). Clinical decision-making skills were assessed before and one month after a three-week VPAR program using a researcher-developed questionnaire. Data were analyzed using R software (version 4.3.3) and the Generalized Estimating Equations (GEE) model to determine the effect of the intervention. The post-test probability of a correct response was 7.34 times greater than the pre-test (p = 0.0684), indicating improved performance likely due to the intervention (p = 0.0018). Additionally, the intervention group had a 3.75 times higher likelihood of correct responses compared to the control group (p = 0.0004), demonstrating the intervention’s positive impact. In conclusion, the VPAR is an effective tool for enhancing clinical decision-making among occupational therapy students.

## Introduction

Clinical training is a cornerstone of healthcare education, requiring diverse and innovative teaching methods to equip students with the practical skills and competencies necessary for professional practice. Traditional lecture-based approaches, while widely used, often fall short in fostering critical thinking, problem-solving, and hands-on clinical skills, which are essential for effective patient care [1]. As healthcare systems evolve, there is a growing demand for educational strategies that are more interactive, flexible, and aligned with the complexities of modern clinical environments [2].This has led to increased interest in simulation-based learning, which bridges the gap between theoretical knowledge and practical application by allowing students to practice in realistic, risk-free setting [3,4].

Among the most promising innovations in medical education are virtual patients (VPs), which have transformed clinical training by providing immersive, scenario-based learning experiences [3–6].VPs enable students to engage in diagnostic reasoning, clinical decision-making, and self-directed learning through interactive case simulations, assessments, and immediate feedback [7,8].This approach not only enhances critical thinking and evidence-based practice but also allows learners to make and learn from mistakes without jeopardizing patient safety [5–8].

Augmented reality (AR) has further expanded the possibilities of simulation-based education by integrating virtual elements into real-world environments, creating highly immersive and interactive learning experiences [9]. AR technology, which utilizes tools such as location tracking, image recognition, and sound detection, has been shown to improve learning outcomes, reduce training costs, and increase educational efficiency [10]. Studies demonstrate that AR-based simulators enhance healthcare education by reducing errors, shortening training time, improving accuracy, and boosting learner motivation and engagement [11,12]. Additionally, AR strengthens cognitive and psychomotor skills, supports knowledge retention, and fosters teamwork and decision-making abilities [13,14].

The integration of virtual patient technology with augmented reality represents a significant advancement in healthcare education. This combination has been shown to enhance learning outcomes by promoting active participation, repeated practice, and hands-on experiences, all of which are critical for long-term knowledge retention [15,16]. For instance, an AR and haptic feedback simulator for pedicle screw placement demonstrated a 15% increase in accuracy and a 50% reduction in performance variability [17]. Similarly, an AR application for remote nursing education on heart failure allowed students to interact with a 3D model of the human heart via mobile devices, achieving comparable effectiveness to traditional video lectures while significantly improving student engagement [18].

While the use of augmented reality (AR)-based virtual patients has gained considerable attention across various educational fields, research within the field of occupational therapy, particularly concerning the Nerves of the upper limbs domain, remains limited [19]. This issue becomes even more critical given the essentiality of continuous learning and repeated practice for the maintenance of practical skills in occupational therapy [20,21]. Within this context, augmented reality-based virtual patient (VPAR) education offers a promising tool to provide sustained, cost-effective, and engaging learning experiences [22].

The conceptual framework of this study is grounded in two theoretical models: Experiential Learning Theory and Cognitive Load Theory. According to Experiential Learning Theory, developed by David Kolb, learning is most effective when the learner actively participates in a cyclical process comprising four stages: concrete experience, reflective observation, abstract conceptualization, and active experimentation [23,24].The VPAR environment facilitates this cycle by positioning students in realistic or simulated clinical scenarios, enabling them to transform theoretical concepts into practical application through observation and feedback.

Furthermore, based on Cognitive Load Theory, working memory capacity is limited, and to optimize learning, instructional design should reduce extraneous cognitive load while enhancing germane cognitive load, with intrinsic cognitive load reflecting the inherent complexity of the instructional content [25–27]. Intrinsic load relates to the inherent difficulty of the subject matter, extraneous load refers to poorly designed content and unnecessary information that must be minimized, and germane load pertains to deep processing and the construction of effective cognitive schemas that should be fostered. Therefore, the VPAR educational program is designed to minimize extraneous load through multimodal presentation and immediate feedback, optimize cognitive processing associated with clinical reasoning, and control intrinsic load via scaffolder scenarios [25–27].

Therefore, this study aims to investigate the effect of augmented reality-based virtual patient training on clinical decision-making skills of occupational therapy students, with a specific focus on upper limb Nerves. By exploring this innovative approach, the study seeks to contribute to the growing body of evidence on the use of AR and virtual patients in healthcare education and provide insights into their potential to transform clinical training.

## Materials and methods

### Study design

This quasi-experimental study utilized a pre-test and post-test design with two groups (intervention and control).

### Participants

This study included undergraduate occupational therapy students enrolled in Occupational Therapy in Physical Disabilities. The intervention group comprised students from Shiraz University of Medical Sciences (School of Rehabilitation), while the control group consisted of students from Arak University of Medical Sciences (School of Rehabilitation).

### Inclusion and exclusion criteria

Eligible participants were occupational therapy students enrolled in ‘Occupational Therapy in Physical Disabilities’ who met these criteria: no prior training in upper limb lesions, provision of signed informed consent, access to reliable internet and an Android device, and experience using mobile educational apps. Students were excluded if they withdrew consent or attended fewer than two training sessions.

### Setting

The study population consisted of under graduate occupational therapy students s who were enrolled in the ‘Occupational Therapy in Physical Disabilities II course affiliated with Shiraz and Arak University of Medical Sciences from January to April 2025.

### Educational intervention

After obtaining the necessary approvals from the university’s research committee, the researchers began the study by providing an overview of the initiative to the participants and obtaining their written consent. Eligible students who agreed to participate in the study signed the informed consent form and completed a pre-test questionnaire to assess their Clinical decision-making. The intervention group received educational content through the VPAR app on their mobile phones, while the control group received conventional method.

### Intervention group

The intervention involved developing educational materials and selecting instructional approaches through a collaboration between an e-learning specialist and an occupational therapy educator with 10–15 years of teaching experience. The content was designed to enhance occupational therapy students’ clinical decision-making skills, particularly in the upper extremity neurological injury curriculum. Course objectives aligned with the latest Occupational Therapy in Physical Disorders standards, and topics remained consistent between the intervention and control groups [28]. Implementation followed a structured approach. The VPAR group participated in three weeks of 30-minute case studies, each introduced with a brief in-class overview and supported by technical and educational assistance. To promote focused learning, one case (Radial, Median, or Ulnar nerve injury) was unlocked per week.

### VPAR application for occupational therapy education

**Fig 1:**
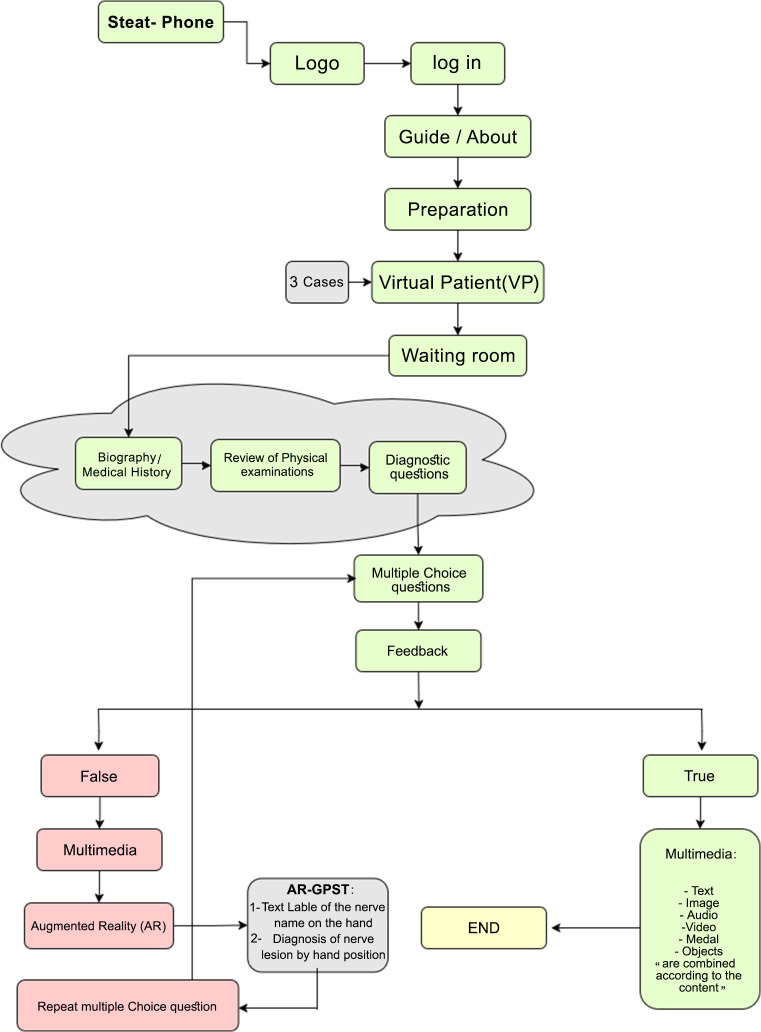
VPAR application learning path (Designed on Diagram.net).

The VPAR application, an educational software tool, was developed to improve occupational therapy students’ the Clinical decision-making by simulating real-world clinical training. Grounded in experiential learning theory and cognitive load theory [23,25]. it integrates virtual patient scenarios with augmented reality (AR) to strengthen anatomical knowledge and clinical reasoning through deliberate practice. The app’s content was reviewed and approved by two occupational therapy experts and two orthopedic specialists before being developed by e-learning specialists following multimedia principles. The final product incorporated audio, text, images, videos, avatars, and gamification elements to create an interactive learning experience. The environment of the VPAR application is shown in Fig 1.

The VPAR application’s core innovations included its hybrid design, combining virtual patients with branching clinical cases, AR for kinesthetic learning through real-time feedback, and gamified competency rewards. By blending these elements, the app provided an immersive, hands-on training tool tailored to the needs of occupational therapy education.

**Fig 2:**
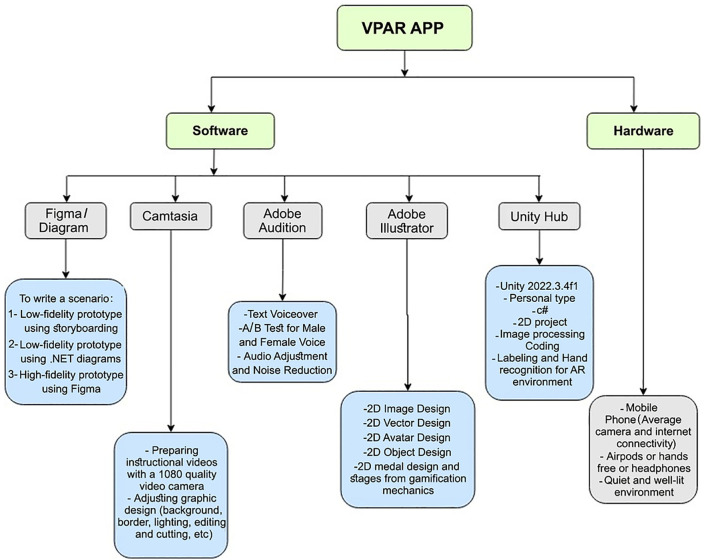
VPAR application hardware and software (Designed on Diagram.net).

The VPAR application was developed using Android mobile phones, iPads, and various software tools, including Unity, Illustrator, Adobe Audition, Camtasia, Diagrams.net, and Figma. Diagrams.net served for the initial prototype, while Figma refined the interface. Voiceovers were recorded by an experienced instructor, and Adobe Audition was used for noise adjustment. Educational videos were filmed using 1080p cameras and edited with Camtasia (Fig 2).

Key interactive features included AR-GPS functionality for real-time hand positioning feedback, gamified assessments with medals and competency-based rewards, and virtual patient scenarios covering Radial, Median, and Ulnar nerve injuries.

**Fig 3:**
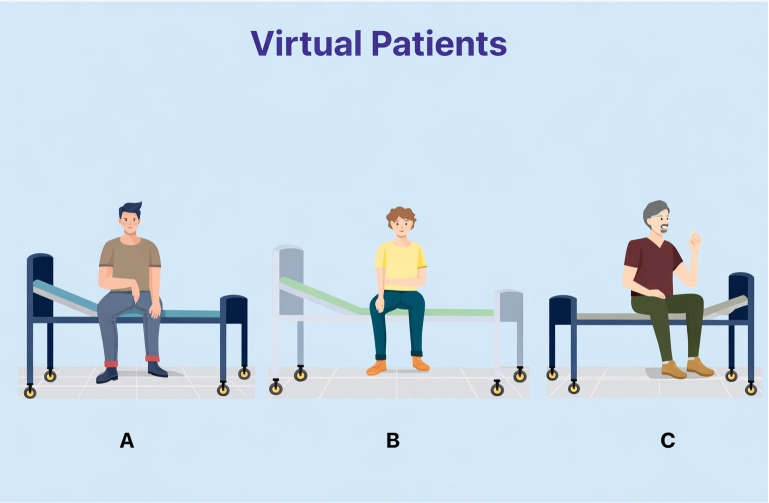
Screenshot of the virtual patient page in the VPAR app.

Through virtual patients, students interacted with clinical scenarios (Fig 3).

**Fig 4:**
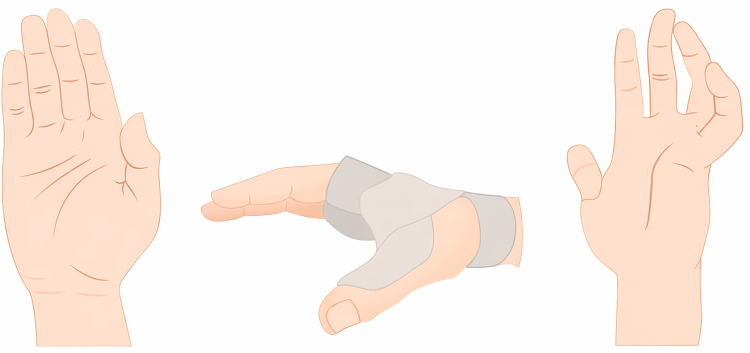
Images Created in Illustrator at 1080p Resolution.

If they chose the right answer, they received multimedia feedback. If wrong, they used their phone’s camera to position their wrist in specific ways to simulate injuries. The app would confirm when the position was correct, allowing for realistic, hands-on learning. Students could not proceed until they correctly completed each step, ensuring they fully understood the material before moving on. This method allowed students to practice repeatedly, make decisions, and learn through experience (Fig 4).

**Fig 5:**
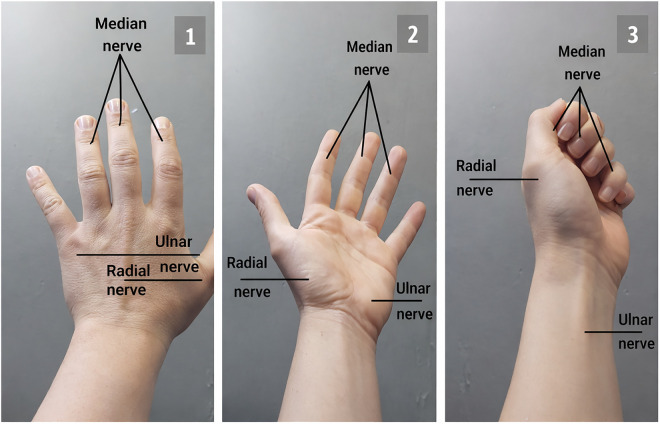
Augmented Reality Identifying Hand Nerves with Text Labeling.

In Fig 5, the nerves of the hand are displayed in an augmented reality environment with text labeling, using the phone’s camera and environment scanning. In image 1, the nerves of the back of the hand are shown; in image 2, the nerves inside the hand; and in image 3, the hand, wrist, and fingers are positioned together, all of which provide feedback for diagnostic questions in the real-world environment (AR).

**Fig 6:**
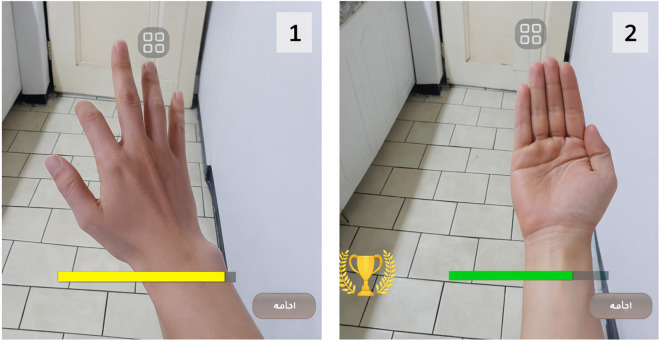
Augmented Reality Identifying Hand Nerves using Gesture Recognition (AR-GPS).

In Fig 6, the median nerve lesion is displayed in the augmented reality environment through hand movement recognition using the phone’s camera and environment scanning. In image 1, the student was unable to position their hand in the form of the median nerve injury, which is why a yellow bar (a gamification element) appears. However, in image 2, the same student correctly positions their hand to reflect the median nerve injury, and the green bar indicates correct learning. In this image, you can also see the trophy symbol, which signifies the accuracy of previous diagnostic questions, and this is another gamification mechanic.

Key interactive features of the VPAR app included AR-GPS functionality for real-time hand positioning feedback, gamified assessments with medals and competency-based rewards, and virtual patient scenarios covering Radial, Median, and Ulnar nerve injuries.

Through virtual patients, students interacted with clinical scenarios. If they chose the right answer, they received multimedia feedback. If wrong, they used their phone’s camera to position their wrist in specific ways to simulate injuries. The app would confirm when the position was correct, allowing for realistic, hands-on learning. Students could not proceed until they correctly completed each step, ensuring they fully understood the material before moving on. This method allowed students to practice repeatedly, make decisions, and learn through experience.

**Fig 7:**
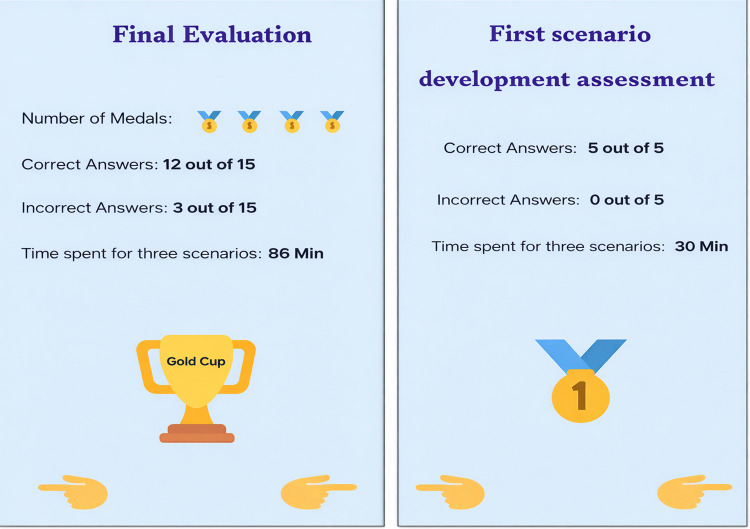
Screenshot of a student’s gamified formative and final assessment.

The app included three virtual patient scenarios, each comprising five multiple-choice questions ranging from diagnosis to treatment. Each scenario included a toolbar that indicated the number of questions as well as whether the responses were correct or incorrect. Students who answered at least three out of five questions correctly earned a medal, with final evaluations awarding a gold cup for three medals and a silver cup for two. To support usability, students received initial app instructions and in-class guidance (Fig 7).

### Control group

The control group participated in three traditional classroom sessions, each lasting 1 hour, for a total of 3 hours of instruction. Students were educated about upper extremity nerve injuries through conventional lecture-based methods supported by PowerPoint presentations. Before the training sessions, students completed a pre-test using a researcher-made questionnaire. The course content was presented by an instructor in three consecutive sessions.

### Sample size & randomization

A total of 44 occupational therapy students enrolled in the Occupational Therapy course at Shiraz and Arak Universities of Medical Sciences were selected through census sampling. Since individual randomization was not feasible in this educational context, cluster randomization was applied at the class level. The class from Shiraz University was assigned to the intervention group (VPAR, n = 24), and the class from Arak University served as the control group (traditional education, n = 20). This method helped prevent contamination between students within the same institution; however, it may introduce potential bias due to institutional differences. To address this, both groups received the same course objectives and teaching standards, and demographic characteristics were collected to ensure baseline comparability. Cluster randomization is a well-established approach in educational and clinical studies when individual randomization is not possible. With an alpha error of 5%, power of 95%, and an effect size of 39%, the required sample size was determined to be 24 participants [29].

### Data collection tools

The Upper Limb Nerve Injury Questionnaire (ULNQ) is a researcher-developed instrument designed to assess students’ clinical diagnostic skills regarding upper limb nerve injuries. To develop this questionnaire, the research team reviewed and extracted relevant questions from validated national examination tests and reputable scientific sources in the field of occupational therapy. Initially, the course instructor selected 25 multiple-choice questions based on real clinical scenarios. These questions underwent validity and reliability assessments, resulting in the final selection of 20 questions with the highest validity and reliability for inclusion in the questionnaire.

This questionnaire is scenario-based was utilized. For questionnaire distribution, the names of students from both groups were obtained from the faculties, and the questionnaires were coded according to this list. The same coding system was used for both assessment phases. At the beginning of the course, a pretest was administered to both the intervention and control groups, and one month after the intervention, the same questionnaire was used again as a posttest. The data collection tool was a researcher-developed questionnaire consisting of two main sections:

- Section One: Demographic information of the students, including age, gender, Marital status, Employment status, and overall GPA.

Section 2 includes specialized scenario-based questions designed to assess students’ diagnostic skills in radial, median, and ulnar nerve injuries. The questionnaire consists of 20 clinical scenarios: 6 related to the radial nerve, 6 to the median nerve, and 8 to the ulnar nerve. Each scenario presents a concise patient history and key physical examination findings, followed by four diagnostic options, only one of which is correct. Scoring is binary, with 1 point for each correct response and 0 for incorrect answers, resulting in a total possible score ranging from 0 to 20. A few sample items are provided in S1 Table to illustrate the structure and scoring approach (Table 1).

**Table 1:** Questionnaire score classification table.

Category	Score range (0–20)	equivalent percentage
great	17 − 20	85–100%
good	13 −16	65–84%
average	9 −12	45–64%
weak	0 - 8	0–44%

This classification is used to ultimately evaluate students’ skills in diagnosing upper limb nerve lesions and is determined based on the percentage of the total score.

- Content Validity

10 experts (4 in Occupational Therapy, 3 in Physical Therapy, and 3 in Orthopedics) evaluated content validity of the questionnaire using Content Validity Index (CVI) and Content Validity Ratio (CVR) methods. Experts rated each question based on relevance, clarity, and simplicity on a 1–4 scale. For CVI, the ratio of experts giving scores of 3 or 4 was calculated. According to Waltz and Bausell, a CVI of 0.79 or higher is acceptable, and 0.70 is acceptable with revisions. For CVR, experts assessed whether each item was “essential” or “non-essential”, and according to Lawshe’s model, a CVR of 0.62 or higher is acceptable when evaluated by 10 experts [30,31]. Overall, by collecting feedback from the scorers, a CVR of 0.92 and a CVI of 0.93 were obtained. Also, evaluation of three CVI subscales – relevance (CVI = 0.89), clarity (CVI = 0.95), and simplicity (CVI = 0.96) – confirmed satisfactory scores for these subscales.

- Reliability

To estimate the reliability of the tool, the internal consistency method using the Kuder-Richardson Formula 20 (KR-20) was applied. After collecting 80 samples, KR-20 was calculated. Since this research aims to evaluate the impact of education using the VPAR application, validating the researcher-made questionnaire is highly significant. Therefore, the overall reliability of the questionnaire was calculated. The Kuder-Richardson index ranges from 0 to 1, with values closer to 1 indicating greater internal consistency among the questions. The expected reliability of a questionnaire is at least 0.70, and a range between 0.80 and 0.90 is considered excellent. Reliability analysis of the questionnaire using the internal consistency method and the Kuder-Richardson coefficient showed an overall reliability of 0.892, which is considered excellent considering the baseline and standard reliability scores.

### Statistical methods

Data from this study were analyzed using R software (version 4.3.3) employing the Generalized Estimating Equations (GEE) model to examine the effect of the intervention on students’ accuracy. The GEE model, appropriate for longitudinal and correlated data, included time (pre-test vs. post-test), group (intervention vs. control), and gender as independent variables, with response accuracy (correct/incorrect) as the outcome measure. An exchangeable correlation structure was specified to account for repeated measurements within participants. This structure assumes that correlations between repeated observations within the same subject are equal and constant. It is one of the most commonly used correlation structures, providing robust and stable parameter estimates even if the true correlation structure deviates slightly from this assumption. Statistical significance was set at p < 0.05.

### Ethics

This study was approved by the local ethics committee of Shiraz University of Medical Sciences (IR.SUMS.REC.1403.324) and coordinated with the relevant faculties. The research team explained the study objectives to participants during an initial phone call, after which the informed consent form was emailed and written informed consent was obtained. Participation was voluntary, and participants were informed of their right to withdraw from the study at any time. To ensure anonymity and confidentiality, no identifying information was collected, all questionnaires were completed anonymously, and a research assistant coded the data to prevent errors during data entry. Only aggregated results were reported. All study procedures were conducted in accordance with relevant ethical guidelines and regulations. To uphold educational fairness, the VPAR application was made available to the control group after completion of the study.

## Results

**Fig 8:**
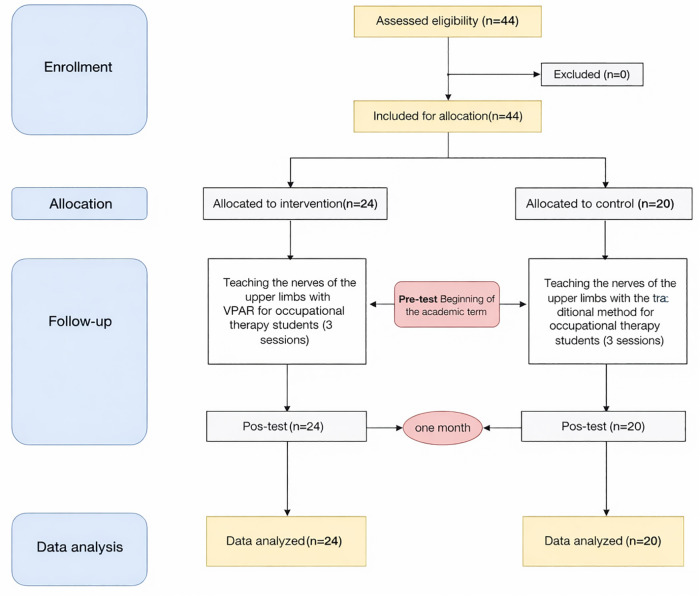
The participants’ recruitment flow diagram.

In this study, a total of 44 students participated, comprising 20 individuals in the control group and 24 in the intervention group (Fig 8).

To assess differences between the two groups across variables such as group assignment (intervention vs. control), time points (pre-test and post-test), age, gender distribution, marital status, employment status, overall GPA, previous semester GPA, or experience of online education year of admission, the Generalized Estimating Equations (GEE) model was employed to analyze learning rates. Participants’ demographic characteristics are shown in Table 2.

**Table 2:** Baseline demographic and academic characteristics of participants.

Characteristic	Control (Arak) (n = 20)	Intervention (Shiraz) (n = 24)	p-value
**Age, years (mean ± SD)**	22.25 ± 1.49	21.92 ± 1.67	0.38¹
**Gender, n (%)**			0.87²
Female	12 (60.0)	15 (62.5)	
male	8 (40.0)	9 (37.5)	
**Marital status, n (%)**			1.00²
Single	17 (85.0)	21 (87.5)	
Married	3 (15.0)	3 (12.5)	
**Employment status, n (%)**			0.74²
Employed	7 (35.0)	7 (29.2)	
Unemployed	13 (65.0)	17 (70.8)	
**Overall GPA (mean ± SD)**	16.23 ± 1.02	15.76 ± 1.17	0.08¹
**Previous semester GPA (mean ± SD)**	16.22 ± 1.08	15.68 ± 1.18	0.11¹
**Experience of online education, n (%)**	20 (100.0)	24 (100.0)	

**¹**Independent t-test; **²**Fisher’s exact test.

Data are presented as mean ± standard deviation for continuous variables and number (percentage) for categorical variables.

Results indicated no statistically significant differences between groups regarding gender, year of admission, age, academic semester, or field of study at the 95% confidence level, confirming baseline comparability. Statistical comparisons were conducted using independent t-tests for continuous variables and Fisher’s exact test for categorical variables. Both groups had a similar proportion of female participants (62.5% in Shiraz vs. 60% in Arak), and most students in each group were single and not employed. All participants had experience with online education (100%). These findings confirm that the groups were demographically and academically comparable at baseline, supporting the internal validity of subsequent analyses (Table 3).

**Table 3:** presents the means and standard deviations (mean ± SD) of pre-test and post-test scores for both control and intervention groups.

Time-Group	mean	SD
Pre-Test. control	0.28	0.449
Post-Test. control	0.585	0.493
Pre-Test. intervention	0.268	0.443
Post-Test. intervention	0.790	0.404

The data indicate a notable improvement in the intervention group, where the mean score increased from 0.27 (SD = 0.44) at pre-test to 0.79 (SD = 0.40) at post-test, demonstrating the effectiveness of the intervention. In contrast, the control group showed a smaller increase in mean scores from 0.28 (SD = 0.45) to 0.58 (SD = 0.49). The standard deviation values reflect the variability of scores within each group and time point. These descriptive statistics, combined with the Generalized Estimating Equations (GEE) results, provide a comprehensive understanding of the intervention’s effect size and its impact over time.

The Diagnostic Decision-Making scores of the intervention and control groups were assessed and contrasted before and one month after the intervention, Generalized Estimating Equations (GEE) model. Specifying the exchangeable correlation structure ensures more accurate standard error estimation and valid inference, enhancing the reliability and interpretability of the findings. (Table 4).

**Table 4:** Comparison of diagnostic decision-making scores between intervention and control groups before and after the intervention.

Variable	Estimate	Std. Error	Wald Statistic	P-value	adjusted OR* (95% CI**)
**Intercept**	−0.683	0.375	3.32	0.0684	0.505 (0.25- 1.02)
**Time (Post-Test vs. Pre-Test)**	1.993	0.64	9.7	**0.0018**	7.34 (2.35-22.95)
**Gender (Male vs. Female)**	−0.708	0.415	2.9	0.0884	0.49 (0.21-1.11)
**Group (Intervention vs. Control)**	1.323	0.373	12.55	**0.0004**	3.75 (1.77-7.95)

*OR (Odds Ratio) refers to the ratio of the odds of an event occurring in one group to the odds of it occurring in another group, while controlling for potential confounding variables.

**The “95% CI” (95% Confidence Interval) indicates the range within which the true odds ratio is expected to fall with 95% confidence, reflecting the precision and reliability of the estimate.

Table 4 presents the comparison of Diagnostic Decision-Making scores between the intervention and control groups, evaluated before and one month after the intervention, analyzed using a Generalized Estimating Equations (GEE) model. The estimates are accompanied by standard errors, Wald statistics, p-values, adjusted odds ratios (ORs), and their corresponding 95% confidence intervals (CIs), which provide a range within which the true OR is expected to lie with 95% certainty.

The time effect (post-test vs. pre-test) shows a significantly higher likelihood of correct responses after the intervention, with an adjusted odd ratio (OR) of 7.34 (95% CI: 2.35–22.95, p = 0.0018). This means that participants were over seven times more likely to answer correctly in the post-test compared to the pre-test, indicating a strong positive impact of the intervention and time on clinical decision-making skills.

The adjusted odds ratio (OR) for male versus female participants was 0.49, with a 95% confidence interval (CI) ranging from 0.21 to 1.11, and a p-value of 0.0884. Since the 95% CI includes 1 and the p-value exceeds the conventional significance threshold of 0.05, this difference is not statistically significant.

The group variable, comparing the intervention to the control group, yielded an adjusted OR of 3.75 (95% CI: 1.77–7.95, p = 0.0004). This indicates that participants in the intervention group were nearly four times more likely to provide correct answers than those in the control group. The confidence interval excludes 1, confirming the statistical significance and robustness of the intervention effect.

## Discussion

This quasi-experimental study clearly demonstrated that augmented reality technology based on virtual patients (VPAR) significantly outperformed traditional educational methods. The results highlight two important aspects: first, students in the VPAR group had a 3.75 times higher likelihood of providing correct diagnostic responses compared to the control group, indicating the effectiveness of this technology relative to traditional instruction. Second, the odds of correct responses increased 7.34-fold from pre-test to post-test among students, reflecting a notable improvement in clinical decision-making accuracy due to the positive influence of the intervention over time. Together, these findings emphasize that VPAR training enhanced clinical decision-making skills by 75% among fifth-semester occupational therapy students, underscoring the profound and meaningful impact of this educational approach on clinical performance. The marked superiority of VPAR lies in its ability to address critical limitations of conventional pedagogy through immersive problem-based learning, real-time feedback, and gamification. Unlike traditional methods, which often rely on passive lectures or static textbooks, VPAR immerses learners in interactive, realistic clinical scenarios. For example, students engaged with virtual patients represented as two-dimensional vectors, diagnosing injuries and adjusting treatments in dynamic environments. This active participation mirrors findings by Mehrotra et al. (2021), who emphasized that augmented reality (AR) simulations enhance engagement by placing learners in decision-making roles. The gamified elements of VPAR—such as earning “medals” for correct decisions or receiving instant color-coded feedback (e.g., green/yellow charts)—create a motivational loop that reinforces learning, a feature absent in routine education [32].

A key advantage of VPAR over traditional training is its capacity to deliver immediate, context-specific feedback. In classroom settings, feedback delays often hinder students’ ability to connect actions with outcomes [33].VPAR circumvents this by evaluating techniques in real time; for instance, when students performed hand movements (e.g., rotating or closing hands) to align with augmented reality overlays, the system provided instant corrections. This aligns with Nifakas et al. (2014), who found that AR-based virtual patients improved clinical reasoning through iterative feedback, enabling learners to refine their approaches dynamically. Such immediacy is critical for procedural mastery [34], as shown by Gasco et al. (2014), where simulation-based spinal surgery training enhanced residents’ technical precision through repetitive, feedback-driven practice. Traditional methods, which lack this interactivity, struggle to replicate the nuanced skill development required for clinical competence [35].

The VPAR educational model integrates Kolb’s experiential learning theory and cognitive load principles to optimize clinical skill acquisition. Students progress through simulated scenarios (concrete experience), immediate feedback (reflective observation), reasoning analysis (abstract conceptualization), and real-world practice via augmented reality (active experimentation) [23,24,36]. By reducing extraneous load through structured multimedia, managing intrinsic load via progressive content, and enhancing germane load through focused decision-making practice [25–27,37], the program promotes effective learning transfer from virtual to clinical settings. Consistent with this theoretical foundation, augmented reality–based virtual patient education led to over 75% improvement in intervention group performance, demonstrating significant enhancement of clinical decision-making skills. These findings further suggest that augmented reality not only deepens understanding but also facilitates the practical application of clinical skills, reinforcing the validity and impact of the educational design [23–27,36,37].

VPAR further bridges gaps in spatial and procedural understanding, areas where conventional methods falter. By leveraging markerless AR technology with GPS-based motion detection, students interact with 3D anatomical models in their physical environment, visualizing how movements affect musculoskeletal structures during rehabilitation [38]. This contrasts sharply with traditional tools like static diagrams or pre-recorded videos, which fail to convey dynamic anatomical relationships. Alexander et al. (2024) similarly highlighted AR’s ability to enhance comprehension of complex medical concepts through interactive environments—a strength particularly evident in occupational therapy, where hands-on skill application is paramount [39].

The accessibility and scalability of VPAR also address systemic limitations of traditional clinical training. Conventional methods depend on resource-intensive simulations labs, standardized patients, or supervised placements, which are often geographically restricted. VPAR’s mobile platform democratizes access, allowing students to practice anytime and anywhere, ensuring consistent exposure to diverse clinical scenarios. This scalability aligns with Sargolzaei et al. (2023), where virtual reality-based coronary artery bypass graft (CABG) training outperformed traditional methods by fostering adaptability in operating room students [38].

While the benefits of VPAR are clear, conflicting studies underscore the importance of design specificity. For example, Stepan et al. (2017) found no knowledge gain with 3D neuroanatomy models compared to textbooks [40], and Herbert et al. (2021) reported similar learning outcomes between AR and video groups [18]. These discrepancies likely arise from differences in educational design: VPAR’s success hinges on its problem-based structure, which requires active decision-making, whereas Herbert’s AR model lacked clinical tasks. Similarly, Stepan’s generic 3D brain model omitted the clinically relevant scenarios central to VPAR, such as injury diagnosis tailored to occupational therapy. The absence of real-time feedback in these studies further highlights how VPAR’s instant evaluation bridges theory and practice, a factor critical to its efficacy.

In conclusion, VPAR’s superiority stems from its synthesis of immersive interactivity, contextual feedback, and scalable accessibility. By simulating real-world clinical pressures and enabling iterative skill refinement, VPAR prepares students for healthcare’s dynamic demands. While technical hurdles remain, the demonstrated improvements in clinical decision-making underscore AR’s transformative potential in modern medical education, offering a compelling alternative to traditional methods constrained by passivity, delayed feedback, and resource limitations.

The study had a relatively small sample size, which may limit the generalizability of the findings. Future studies should aim to include a larger sample size to better understand the impact of the AR-based virtual patient training on occupational therapy students’ clinical decision-making.

In this study, a significant intracluster correlation was observed, indicating the presence of similarities among members within the same cluster; however, differences also existed between clusters. Given the limited number of clusters, this issue could lead to reduced statistical power in the analyses. The pre-determined cluster design of the study, combined with a reduced sample size per cluster, resulted in limitations in the analysis and interpretation of the results. Although the use of the GEE statistical model, which defines responses within each cluster, attempted to control these correlations, the reduction in statistical power caused by the cluster structure remains one of the main limitations that should be addressed in future studies.

Although the selected sample size met the requirements for necessary statistical analyses, the small sample dimensions, which are typically common in augmented reality and virtual patient-based educational studies, may limit the generalizability of the results to larger populations. Conducting the study in one country and only two universities reduces the transferability of the findings to different cultural and educational settings because the effectiveness of novel educational technologies such as augmented reality is influenced by diverse technological and educational contexts. The study follow-up was limited to a short one-month period, allowing only the evaluation of short-term learning outcomes. Considering previous research evidence, the effects of simulation-based skills may decline over time without reinforcement, thus long-term evaluations are essential to understand the sustainability of these outcomes. Furthermore, focusing the study on occupational therapy students limits the applicability of the findings to other health professions that have different cognitive and procedural constructs.

Finally, relying solely on quantitative measures in this research diminished the depth of insight into learners’ experiences, motivation, and usability—dimensions that are typically better understood and explored through qualitative data. Despite these common limitations in quasi-experimental educational research, our findings highlight the strong potential of augmented reality-based virtual patient training. In this version, quantitative indices including CVI and CVR were used, indicating acceptable item quality. However, construct validity of the ULNQ questionnaire was not assessed in this study, and it is recommended that future studies report construct validity alongside the two validity indices CVI and CVR. Additionally, employing qualitative methods such as interviews or focus groups could provide deeper insights into experiences, perceptions, and factors affecting the effectiveness of this educational approach.

Moreover, larger samples, longer follow-ups, and mixed-method approaches could further strengthen the evidence base. Addressing these limitations in future research can enable a more comprehensive understanding of the potential benefits and best practices for implementing augmented reality-based virtual patient training in healthcare education, ultimately enhancing clinical decision-making skills of future healthcare professionals.

The VPAR system can be integrated as a supplementary clinical reasoning module within courses related to upper-limb neurology, enabling students to engage in repeated diagnostic practice through immersive, augmented reality–based simulations. Educational research consistently demonstrates that the integration of structured simulation, when aligned with course objectives, significantly enhances learners’ clinical reasoning and performance through deliberate practice, immediate feedback, and experiential learning [5,20].The design of VPAR, which incorporates experiential learning theory and cognitive load optimization, provides a suitable educational model that can be embedded in weekly laboratory sessions, preclinical preparatory programs, or as a self-directed mobile learning platform to reinforce clinical decision-making both inside and outside the classroom. By including VPAR as a formative assessment tool, instructors can monitor learner progress, identify areas requiring improvement, and deliver personalized feedback. Furthermore, the mobile-based architecture of VPAR supports scalability and accessibility, allowing programs with limited laboratory resources to offer high-quality simulation experiences at low cost, an advantage highlighted in recent simulation literature [19].

## Conclusion

This quasi-experimental study demonstrates that the integration of augmented reality (AR) and virtual patients (VP) into occupational therapy education significantly enhances students’ clinical decision-making skills. By combining interactive 2D simulations with immersive real-world AR environments, this hybrid educational model fosters authentic, experiential learning scenarios that deepen practical skill development and cognitive engagement in realistic clinical contexts. The results reveal a statistically significant increase in the probability of correct clinical decisions among students exposed to this innovative training, providing robust evidence of the effectiveness and transformative potential of AR-based virtual patient (VPAR) training. This integration not only immerses learners in realistic scenarios but also facilitates the transfer of knowledge to real-world clinical practice, thus better preparing future occupational therapists for complex healthcare challenges. From an educational policy and curriculum development perspective, these findings emphasize the critical importance of systematically embedding AR-based tools within occupational therapy programs. Such integration promotes active, learner-centered education while overcoming traditional challenges related to limited resources and geographic barriers by providing scalable and accessible simulation experiences. Institutions are encouraged to support the adoption of AR technologies through comprehensive policies encompassing curriculum redesign, faculty training, and infrastructure investment, ensuring sustained, equitable access. Moreover, promoting accessibility and sustainability through mobile, flexible AR applications aligns with contemporary instructional design principles, including effective cognitive load management and scaffolding of learning activities. This approach supports continuous, lifelong learning for healthcare professionals, enabling adaptability in evolving clinical environments.

In conclusion, AR-based virtual patient training represents a practical, scalable, and evidence-based approach to augment clinical education and decision-making in occupational therapy. It holds significant implications for educational policy and curriculum innovation aimed at preparing students to meet the demands of modern healthcare with enhanced competence, confidence, and readiness.

## Supporting information

S1 Table Expanded ULNQ Sample Items and Scenarios. (DOCX)
