# Supplementary material for: Investigating the effect of augmented reality-based virtual patient training on occupational therapy students’ clinical decision-making: A quasi-experimental study
Source: PLoS One. 2026 Feb 4;21(2):e0340759. doi: 10.1371/journal.pone.0340759 (PMC12872011; doi:10.1371/journal.pone.0340759)
Supplement: S1 Table — (DOCX) [file pone.0340759.s001.docx]

| Detailed Scenario | Question | Options | Correct Answer | Scoring |
| --- | --- | --- | --- | --- |
| Ms. Fatemeh Esmaeili, 45, is a professional tailor who has spent more than 20 years performing repetitive hand movements, including precision stitching and fine needlework. Over the past month, she has experienced progressive numbness, tingling, and reduced sensation in the little finger and the ulnar half of the ring finger on her dominant left hand. She reports difficulty holding needles and frequently dropping small objects. Physical examination reveals reduced light touch and pinprick sensation in the ulnar digits, along with mild weakness in finger adduction. Based on these findings, the clinician suspects an ulnar nerve lesion. | What is the sensory distribution of the ulnar nerve in the hand? | A) Sensation of the center of the palm B) Sensation of the distal phalanges of all digits C) Sensation in parts of the thenar eminence and dorsal proximal phalanx of three and a half fingers D) Little finger and ulnar half of the ring finger | D | 1 = correct, 0 = incorrect |
| Ms. Sara Yazdi, 27, works long hours as a digital illustrator, frequently maintaining sustained wrist flexion and repetitive finger movements. Over several weeks, she developed wrist pain, numbness in the thumb and index finger, and difficulty performing precise drawing motions. On examination, she shows decreased thumb opposition strength and reduced sensation in the radial three and a half digits. Special tests including Phalen’s test reproduce her symptoms. She is diagnosed with median nerve injury at the wrist level. | Which muscles are affected in median nerve injury at the wrist? | A) Pronator Teres, Abductor Pollicis Brevis (APB), Flexor Pollicis Brevis (FPB), Opponens Pollicis B) Abductor Pollicis Brevis (APB), Flexor Pollicis Brevis (FPB), Opponens Pollicis, and the first two lumbricals C) Flexor Digitorum Superficialis (FDS) and Flexor Digitorum Profundus (FDP) D) Flexor Pollicis Longus (FPL), Abductor Pollicis Brevis (APB), Flexor Pollicis Brevis (FPB), Opponens Pollicis | B | 1 = correct, 0 = incorrect |
| Mr. Nader Safaei, 42, a skilled carpenter, sustained a fall at work causing forceful wrist extension injury. Since then, he has difficulty extending his wrist and fingers and notices abnormal finger flexion during passive wrist movements. During evaluation, the therapist explains the tenodesis effect—a passive mechanism in which wrist extension produces finger flexion and wrist flexion produces finger extension. This movement is diminished in Mr. Safaei. Motor testing shows weakness in wrist and finger extensors, and sensory testing reveals deficits on the dorsal radial aspect of the hand. These findings suggest a radial nerve injury. | Injury to which hand nerve impairs the tenodesis movement? | A) Median nerve B) Ulnar nerve C) Musculocutaneous nerve D) Radial nerve | D | 1 = correct, 0 = incorrect |
